# Nitrates as a Marker of Multiple Co-morbidities and Increased Mortality in Patients Undergoing Percutaneous Coronary Intervention (PCI)

**DOI:** 10.7759/cureus.23520

**Published:** 2022-03-26

**Authors:** Neil Yager, Sunjeev Konduru, Mikhail Torosoff

**Affiliations:** 1 Cardiology, Albany Medical College, Albany, USA; 2 Department of Pharmacy, Albany Medical College, Albany, USA

**Keywords:** chronic stable angina, cardio vascular disease, stable ischemic heart disease, cad: coronary artery disease, primary percutaneous coronary intervention (pci)

## Abstract

Background

Notwithstanding the guideline endorsement of various anti-anginal medications, there is a paucity of data on whether one anti-anginal regimen or medication is superior to another. It is also unknown how anti-anginal medications affect outcomes of elective percutaneous coronary intervention (PCI). To fill this knowledge gap, we investigated an association between commonly used anti-anginal medications and elective PCI outcomes in stable ischemic heart disease (SIHD) patients.

Methods

Using the New York State's (NYS) PCI Reporting System, we reviewed data on 33,568 consecutive patients who underwent non-emergent PCI in 2015. The primary endpoint of this study was all-cause in-hospital mortality.

Results

Regardless of the combination therapy of nitrates with any other non-nitrate anti-anginal therapy, including beta-adrenergic blockers (BB) and/or calcium channel blockers (CCB), nitrate treatment continued to be associated with significantly increased post-elective PCI mortality.

Conclusions

In this large, all-inclusive state-wide contemporary cohort study of SIHD patients, treatment with nitrates, but not beta-blockers, calcium channel blockers, or ranolazine, was associated with increased post-PCI mortality. Utilization of nitrate therapy is likely reflective of advanced disease burden rather than directly related to the specific medication intolerance. Additional studies investigating optimal anti-anginal medical therapy on PCI outcomes are warranted.

## Introduction

Most patients with coronary arteriosclerosis have stable ischemic heart disease (SIHD) [1]. To improve SIHD outcomes, 2002 Chronic Stable Angina Guideline and 2007 Focused Update of the American College of Cardiology (ACC)/American Heart Association (AHA) 2002 Guideline for the Management of Patients With Chronic Stable Angina, recommended optimal medical therapy (OMT), including anti-platelet agents, beta-adrenergic antagonists, angiotensin-converting enzyme (ACE) inhibitors or angiotensin receptor blockers (ARBs), and statins [2,3]. With regards to anti-anginal SIHD therapy, guidelines emphasize patient and comorbidity-specific approaches [4]. Recent 2019 European guidelines for the diagnosis and management of chronic coronary syndromes advocate initial therapy with beta-adrenergic blockers and calcium-channel blockers, reserving nitrates and other agents as second-line therapy [5].

Of the commonly used anti-anginal SIHD medications, nitrates were the first medications widely used in the treatment of SIHD related angina since the last century [6-8]. However, while effective in controlling angina, nitrates lack survival benefit, which is the impetus behind the evidence-based OMT [7,9]. With the advent and improved use of OMT, nitrate utilization has decreased significantly and is now primarily limited to patients whose symptoms are not well controlled on evidence-based standard medical therapy aided with revascularization [8].

Notwithstanding the guideline endorsement of various anti-anginal medications, there is a paucity of data on whether one anti-anginal regimen or medication is superior to another [6,10]. It is tempting to speculate that more advanced coronary artery atherosclerosis requires more intense anti-anginal therapy, and thus more intense anti-anginal therapy may be associated with worse elective percutaneous coronary intervention (PCI) outcomes, but this has not been conclusively proven. Furthermore, regarding nitrate therapy, evidence of mortality benefit is lacking, and some data even suggests possible harm from using nitrates in SIHD patients [11-13]. It is also unknown how anti-anginal medications affect outcomes of elective PCI. To fill this knowledge gap, we investigated an association between commonly used anti-anginal medications and elective PCI outcomes in SIHD patients. 

## Materials and methods

Using the New York State's (NYS) PCI Reporting System, we reviewed data on 33,568 consecutive patients who underwent non-emergent PCI in 2015. Patients and public involvement were not present in this study. The New York State's PCI Reporting System collects information from all PCIs performed in nonfederal hospitals in New York State. The Reporting System database includes baseline clinical information, procedural data, and in-hospital post-procedural outcomes. Submitted data underwent intensive quality control, including cross-referencing with the NYS Statewide Planning and Research Cooperative System. This study included all consecutive patients who underwent PCI between January 1, 2015, and December 31, 2015. Patients reported to be in shock or with acute coronary syndromes within 24 hours of the reported index PCI, and patients with prior coronary artery bypass surgery were excluded from the study cohort.

Baseline demographic, medical treatment, and procedural data were collected. The following continuous variables were transformed into categories as follows: 1) age was classified according to the decade of life with all patients younger than 50 years of age were grouped into a single age category, and all patients older than 90 years of age were grouped into the single age category; 2) body mass index was grouped as underweight BMI of <18.5 kg/m^2^, normal BMI of 18.5-24.9 kg/m^2^, overweight BMI of 25-29.9 kg/m^2^, and obese BMI of 30 kg/m^2^ or more; 3) left ventricular ejection fraction was categorized as less than 35%, 35-44%, 45-54%, and 55% and above; 4) renal function was classified by the reported glomerular filtration rate (GFR): GFR 60 ml/min and higher versus GFR less than 60 ml/min.

As recorded in the PCI Reporting System, coronary atherosclerosis involvement was classified according to the angiographic presence of >50% obstruction of the left main (LM) trunk, proximal left anterior descending artery, mid or distal left anterior descending or major diagonal, right coronary artery or posterior descending artery, circumflex artery, or large marginal artery. Patients were grouped according to the presence of a single, two-vessel, three-vessel not including LM, and a separate category of LM involvement with any other disease.

The primary endpoint of this study was all-cause in-hospital mortality. To preserve the intention to treat approach, only baseline clinical, reported medication treatment, and angiographic data were available before the PCI was utilized, while PCI-related procedural factors, including PCI outcomes, were not factored in.

Descriptive analyses were performed with chi-square and t-test or analysis of variance testing, as appropriate, to determine statistically significant differences in mortality between categories and values of individual variables.

To quantify associations between medical treatment, coronary anatomy, clinical comorbidities, and the primary outcome, logistic regression models were constructed to calculate odds ratios with 95% confidence intervals. All models were constructed using a stepwise logistic regression that retained significant independent predictors of in-hospital mortality. Clinical variables tested included age, gender, body mass index, current congestive heart failure, malignant ventricular arrhythmia, diabetes mellitus, chronic obstructive pulmonary disease, left ventricular (LV) ejection fraction, kidney function, pre-procedural medications, and coronary artery anatomy. The concordance C-statistic was used to measure the discrimination of the model. All analyses were performed using StatView 5.0 (SAS Institute, Cary, USA). A p-value of less than 0.05 was considered statistically significant for all analyses.

## Results

The study cohort included 33,568 consecutive patients who underwent non-emergent PCI. Baseline patient characteristics and corresponding in-hospital mortality are presented in Table 1. Advanced age, decreased body mass index, decreased LV ejection fraction, histories of stroke, peripheral vascular disease (PVD), pre-PCI congestive heart failure (CHF), ventricular arrhythmia, chronic obstructive pulmonary disease (COPD), diabetes, kidney dysfunction, and complex coronary anatomy were associated with increased post-elective PCI mortality (Table 1). In addition, treatment with nitrates, but not beta-adrenergic blockers, calcium channel blockers, or ranolazine, was also associated with increased mortality. Nitrate-treated patients represented 18% of the study population and experienced 0.535% post-PCI death rate, as compared to 0.299% (p=0.004) death rate in subjects not reported to be treated with nitrates.

**Table 1:** Prevalence and in-patient mortality rates in PCI according to demographic factors, comorbidities, and anti-anginal medications PCI - percutaneous coronary intervention; TIA - transient ischemic attack; CHF - congestive heart failure; COPD - chronic obstructive pulmonary disease; eGFR - estimated glomerular filtration rate; LM - left main; CAD - coronary artery disease

Risk factor	Cases (n)	Prevalence (%)	Mortality rate (%)	p-value
All patients	33,568	100.00	0.343	
Age				
<50 years old	2,718	8.097	0.037	<0.0001
50-59 years old	7,712	22.974	0.052	
60-69 years old	10,811	32.206	0.231	
70-79 years old	8,128	24.214	0.381	
80-89 years old	3,822	11.386	1.230	
≥90 years old	377	1.123	1.857	
Gender				
Female	10,599	31.575	0.434	0.052
Male	22,969	68.425	0.300	
Weight				
Underweight, BMI <18.5 kg/m^2^	313	0.932	2.875	<0.0001
Normal, BMI 18.5-24.9 kg/m^2^	7,010	20.883	0.613	
Overweight, BMI 25-29.9 kg/m^2^	12,417	36.991	0.250	
Obese, BMI ≥ 30 kg/m^2^	13,828	41.194	0.231	
Left ventricular ejection fraction				
<35%	3,104	9.247	1.160	<0.0001
35-44%	2,777	8.273	0.612	
45-54%	5,954	17.737	0.386	
>55%	21,733	64.743	0.179	
Stroke				
History of stroke or TIA	2,557	7.617	0.782	<0.0001
No history of stroke or TIA	31,011	92.383	0.306	
Peripheral vascular disease				
History of peripheral vascular disease	2,769	8.249	0.831	<0.0001
No history of peripheral vascular disease	30,799	91.751	0.299	
Current CHF				
Current CHF	2,317	6.902	2.287	<0.0001
No current CHF	31,251	93.098	0.198	
Malignant ventricular arrhythmia				
Malignant ventricular arrhythmia	120	0.357	5.0	<0.0001
No malignant ventricular arrhythmia	33,448	99.643	0.326	
COPD				
History of COPD	2,139	6.372	1.216	<0.0001
No history of COPD	31,429	93.628	0.283	
Diabetes mellitus				
History of diabetes mellitus	13,725	40.887	0.423	0.037
No history of diabetes mellitus	19,843	59.113	0.287	
Kidney function				
eGFR <60 ml/min	9,050	27.2	0.757	<0.0001
eGFR ≥60 ml/min	24,383	72.8	0.180	
Coronary anatomy				
1-vessel obstructive disease excluding LM	14,748	43.935	0.136	<0.0001
2-vessel obstructive disease excluding LM	11,891	35.424	0.353	
3-vessel obstructive disease excluding LM	6,342	18.893	0.536	
LM disease with any other obstructive CAD	587	1.749	3.237	
Beta-adrenergic blocker treatment				
Beta-adrenergic blocker	24,187	72.054	0.351	0.656
No beta-adrenergic blocker	9,381	27.946	0.320	
Calcium-channel blocker treatment				
Calcium-channel blocker	9,504	28.313	0.274	0.174
No Calcium-channel blocker	24,064	71.687	0.370	
Nitrate treatment				
Nitrate	6,172	18.3887	0.535	0.004
No nitrate	27,396	81.613	0.299	
Ranolazine treatment				
Ranolazine	2,083	6.205	0.144	0.109
No ranolazine	31,485	93.795	0.356	

**Figure 1:**
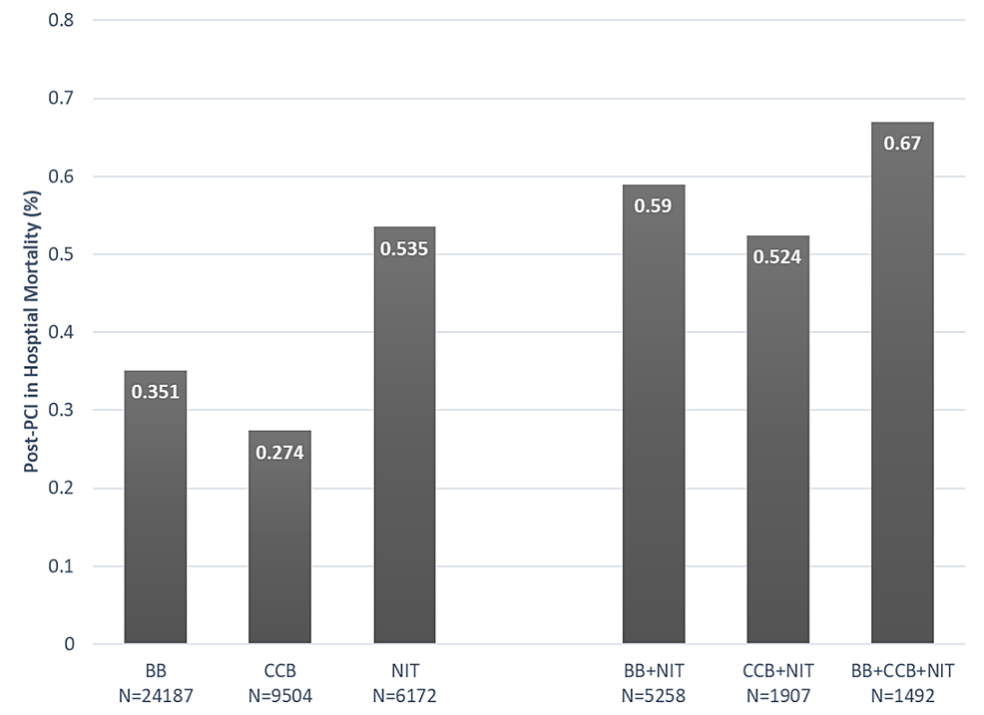
Association between medical therapy and mortality after elective PCI PCI - percutaneous coronary intervention; BB - beta-adrenergic blockers; CCB - calcium channel blockers; NIT - nitrates

Regardless of the combination therapy of nitrates with any other non-nitrate anti-anginal therapy, including beta-adrenergic blockers (BB) and/or calcium channel blockers (CCB), nitrate treatment continued to be associated with significantly increased post-elective PCI mortality (Figure 1). The highest mortality was observed in patients treated with a combination of nitrates, BB, and CCB. Ranolazine was used least frequently, alone or in combination with other medications. Because of small group sizes and low death rate in ranolazine-treated patients (only three deaths were reported), combination therapy with ranolazine was not compared to other combination therapy groups.

The further analysis concentrated on patient characteristics and outcomes associated with nitrate utilization. Treatment with nitrates was more common in females and patients with multiple comorbidities, including advanced age, decreased LV ejection fraction, histories of stroke, PVD, CHF, ventricular arrhythmia, COPD, diabetes, kidney dysfunction, and more complex coronary anatomy (Table 2). Additionally, within each comorbidity group (e.g., advanced age, decreased LV ejection fraction, history of stroke, peripheral vascular disease, congestive heart failure, diabetes, and COPD), treatment with nitrates was associated with increased post-elective PCI mortality. Furthermore, the addition of nitrates to any other anti-anginal therapy effectively doubled post-elective PCI mortality: 0.590 in nitrates and BB vs. 0.285 in BB only groups (p=0.001), 0.524 in nitrates and CCB vs. 0.211 in CCB only groups (p=0.019).

**Table 2:** Utilization of nitrate anti-anginal therapy and comorbidities TIA - transient ischemic attack; CHF - congestive heart failure; COPD - chronic obstructive pulmonary disease; eGFR - estimated glomerular filtration rate; LM - left main; CAD - coronary artery disease † p<0.05 for between with and without Rx use comparisons ‡ p<0.01 for between with and without Rx use comparisons

Risk factor	Nitrate-treated patients (n)	Prevalence of nitrate treatment (%)	Mortality rate (%)		p-value
			Nitrate-treated patients	Patients not on nitrates	
All patients	6,172	18.387	0.535	0.299	0.0043
Age					
<50 years old	464	17.071	0.216	0	0.0275
50-59 years old	1,386	17.972	0	0.063	0.3491
60-69 years old	1,842	17.038	0.326	0.212	0.354
70-79 years old	1,597	19.648	0.501	0.352	0.3873
80-89 years old	794	20.774	2.015	1.024	0.0241
≥90 years old	89	23.607‡	2.247	1.736	0.7549
Gender					
Female	2,216	20.9	0.496	0.418	0.6154
Male	3,956	17.2‡	0.556	0.247	0.0012
Weight					
Underweight, BMI <18.5 kg/m2	49	16.7	4.082	2.652	0.5822
Normal, BMI 18.5-24.9 kg/m2	1,253	17.9	0.479	0.643	0.5009
Overweight, BMI 25-29.9 kg/m2	2,144	17.3	0.56	0.185	0.0016
Obese, BMI ≥ 30 kg/m2	2,726	19.7‡	0.477	0.171	0.0029
Left ventricular ejection fraction					
<35%	617	19.9	1.621	1.045	0.2322
35-44%	573	20.6	0.873	0.544	0.3697
45-54%	1,063	17.9	0.376	0.388	0.9538
>55%	3,919	18.0‡	0.357	0.14	0.0037
Stroke					
History of stroke or TIA	619	24.2	0.808	0.774	0.9338
No history of stroke or TIA	5,553	17.9‡	0.504	0.263	0.0032
Peripheral vascular disease (PVD):					
History of PVD	693	25	1.299	0.674	0.1169
No history of PVD	5,479	17.8‡	0.438	0.269	0.0371
Current CHF					
Current CHF	566	24.4	2.12	2.342	0.7594
No current CHF	5,606	17.9‡	0.375	0.16	0.0011
Malignant ventricular arrhythmia (VT)					
Malignant VT	18	15	5.556	4.902	0.9066
No VT	6,154	18.4	0.52	0.282	0.0031
COPD					
History of COPD	532	24.9	1.504	1.12	0.4839
No history of COPD	5,640	17.9‡	0.443	0.248	0.0125
Diabetes mellitus					
History of diabetes mellitus	2,922	21.3	0.684	0.352	0.0139
No history of diabetes mellitus	3,250	16.4‡	0.4	0.265	0.1891
Kidney function					
eGFR <60 ml/min	2,179	23.9	1.056	0.663	0.065
eGFR ≥60 ml/min	3,988	16.4‡	0.251	0.167	0.2527
Coronary anatomy					
1-vessel CAD excluding LM	2,510	17	0.12	0.139	0.81
2-vessel CAD excluding LM	2,214	18.6	0.452	0.331	0.3867
3-vessel CAD excluding LM	1,319	20.8	1.137	0.378	0.0008
LM disease and any other CAD	129	22.0‡	3.876	3.057	0.6424
Beta-adrenergic blocker treatment					
Beta-adrenergic blocker	5,258	21.739	0.59	0.285	0.001
No beta-adrenergic blocker	914	9.743‡	0.219	0.331	0.5693
Calcium-channel blocker treatment					
Calcium-channel blocker	1,907	20.065	0.524	0.211	0.019
No calcium-channel blocker	4,265	17.724‡	0.539	0.333	0.0445
Ranolazine treatment					
Ranolazine	676	32.453	0.296	0.071	0.2053
No ranolazine	5,496	17.456‡	0.564	0.312	0.0043

In univariate logistic regression analysis, in addition to nitrate treatment, other important predictors of increased post-PCI mortality were increased atherosclerosis burden, advanced age, decreased body mass index, decreased LV ejection fraction, histories of stroke, PVD, CHF, ventricular arrhythmia, COPD, diabetes, and kidney dysfunction (Table 3).

**Table 3:** Univariate logistic regression predictors of in-patient post-PCI mortality PCI - percutaneous coronary intervention; TIA - transient ischemic attack; CHF - congestive heart failure; COPD - chronic obstructive pulmonary disease; eGFR - estimated glomerular filtration rate; LM - left main

N=33,568	Mortality rate = 0.343%		
Risk factor	Coefficient	p-value	OR (95% CI for OR)
Coronary anatomy, as compared to any 1-vessel obstructive disease excluding LM			
2-vessel disease excluding LM	0.959	0.0004	2.610 (1.532-4.448)
3-vessel disease excluding LM	1.379	<0.0001	3.969 (2.283-6.901)
LM and any other disease	3.204	<0.0001	24.633 (13.073-46.415)
Age, as compared to patients younger than 50 years old			
50-59 years old	0.344	0.7587	1.410 (0.157-12.623)
60-69 years old	1.840	0.0712	6.298 (0.853-46.505)
70-79 years old	2.342	0.0212	10.402 (1.419-76.250)
80-89 years old	3.521	0.0005	33.828 (4.663-245.374)
≥90 years old	3.940	0.0002	51.403 (6.305-419.052)
Weight, as compared to normal BMI 18.5-24.9 kg/m^2^			
Underweight, BMI <18.5 kg/m^2 ^	1.568	<0.0001	4.797 (2.317-9.930)
Overweight, BMI 25-29.9 kg/m^2^	-0.903	0.0001	0.406 (0.255-0.644)
Obese, BMI ≥ 30 kg/m^2^	-0.979	<0.0001	0.376 (0.238-0.594)
Left ventricular ejection fraction, as compared to normal ejection fraction >55%			
<35%	1.876	<0.0001	6.527 (4.143-10.284)
35-44%	1.231	<0.0001	3.426 (1.936-6.065)
45-54%	0.769	0.0035	2.157 (1.287-3.614)
Stroke history	0.942	0.0001	2.565 (1.581-4.162)
Peripheral vascular disease history	1.028	<0.0001	2.796 (1.767-4.422)
Current CHF	2.466	<0.0001	11.776 (8.141-17.035)
Malignant ventricular arrhythmia	0.430	<0.0001	16.098 (6.934-37.374)
COPD history	1.466	<0.0001	4.333 (2.793-6.722)
Diabetes mellitus history	0.387	0.0381	1.473 (1.021-2.125)
Kidney dysfunction, eGFR <60 ml/min	1.439	<0.0001	4.217 (2.887-6.161)
Nitrate therapy	0.583	0.0048	1.791 (1.194-2.685)

In multivariate logistic regression analysis, increased atherosclerosis burden, advanced age, decreased body mass index, decreased LV ejection fraction, histories of CHF, ventricular arrhythmia, COPD, diabetes, and kidney dysfunction continued to be associated with increased post-elective PCI mortality (Table 4); while stroke, PVD, and nitrate therapy were not important predictors.

**Table 4:** Multivariate logistic regression prediction model of in-patient post-PCI mortality, including effects of nitrates PCI - percutaneous coronary intervention; CHF - congestive heart failure; COPD - chronic obstructive pulmonary disease; eGFR - estimated glomerular filtration rate; LM - left main * variables eliminated from the logistic regression analysis presented in Table 5

N=33,568	Mortality rate = 0.343%		
Risk factor	Coefficient	p-value	OR (95% CI for OR)
Coronary anatomy, as compared to any 1-vessel obstructive disease excluding LM			
2-vessel disease excluding LM	0.854	0.0023	2.349 (1.357-4.066)
3-vessel disease excluding LM	0.924	0.0018	2.518 (1.410-4.499)
LM and any other disease	2.104	<0.0001	8.199 (4.166-16.137)
Age, as compared to patients younger than 50 years old			
50-59 years old*	0.190	0.8653	1.209 (0.135-10.855)
60-69 years old*	1.413	0.1675	4.108 (0.552-30.560)
70-79 years old*	1.575	0.1237	4.829 (0.650-35.870)
80-89 years old	2.430	0.0173	11.359 (1.535-84.048)
≥90 years old	2.699	0.0130	14.866 (1.765-125.186)
Weight, as compared to normal BMI 18.5-24.9 kg/m^2^			
Underweight, BMI <18.5 kg/m^2 ^	1.285	0.0010	3.615 (1.679-7.785)
Overweight, BMI 25-29.9 kg/m^2^	-0.632	0.0100	0.532 (0.329-0.860)
Obese, BMI ≥ 30 kg/m^2^	-0.605	0.0173	0.546 (0.332-0.899)
Left ventricular ejection fraction, as compared to normal ejection fraction >55%			
<35%	0.722	0.0064	2.059 (1.226-3.460)
35-44%	0.319	0.3116	1.376 (0.741-2.553)
45-54%	0.536	0.0478	1.710 (1.005-2.908)
Stroke history*	0.242	0.3595	1.273 (0.759-2.136)
Peripheral vascular disease history*	0.152	0.5428	1.165 (0.713-1.902)
Current CHF	1.386	<0.0001	3.998 (2.593-6.165)
Malignant ventricular arrhythmia	2.212	<0.0001	9.131 (3.658-22.795)
COPD history	0.807	0.0008	2.241 (1.397-3.597)
Diabetes mellitus history	0.467	0.0224	1.596 (1.068-2.384)
Kidney dysfunction, eGFR <60 ml/min	0.450	0.0344	1.569 (1.034-2.382)
Nitrate therapy*	0.239	0.2738	1.269 (0.828-1.946)

When adjusted for comorbidities, complex coronary anatomy, advanced age, decreased body mass index, decreased LV ejection fraction, CHF, ventricular arrhythmia, COPD, diabetes, and kidney dysfunction remained important predictors of increased post-PCI mortality (Table 5).

**Table 5:** Multivariate adjusted logistic regression prediction model of in-patient post-PCI mortality PCI - percutaneous coronary intervention; CHF - congestive heart failure; COPD - chronic obstructive pulmonary disease; eGFR - estimated glomerular filtration rate; LM - left main

N=33,568	Mortality rate = 0.343%		
Risk factor	Coefficient	p-value	OR (95% CI for OR)
Coronary anatomy, as compared to any 1-vessel obstructive disease excluding LM			
2-vessel disease excluding LM	0.886	0.0015	2.425 (1.401-4.196)
3-vessel disease excluding LM	0.976	0.0009	2.654 (1.488-4.735)
LM and any other disease	2.183	<0.0001	8.873 (4.516-17.432)
Age greater than 80 years old	1.139	<0.0001	3.124 (2.072-4.710)
Weight, as compared to normal BMI 18.5-24.9 kg/m^2^			
Underweight, BMI <18.5 kg/m^2 ^	1.308	0.0008	3.697 (1.722-7.940)
Overweight, BMI 25-29.9 kg/m^2^	-0.659	0.0071	0.517 (0.320-0.836)
Obese, BMI ≥ 30 kg/m^2^	-0.690	0.0063	0.502 (0.306-0.823)
Left ventricular ejection fraction, as compared to normal ejection fraction >55%			
<35%	0.713	0.0073	2.040 (1.211-3.437)
35-44%	0.321	0.3089	1.379 (0.743-2.560)
45-54%	0.512	0.0583	1.669 (0.982-2.835)
Current CHF	1.445	<0.0001	4.242 (2.745-6.556)
Malignant ventricular arrhythmia	2.287	<0.0001	9.843 (3.965-24.438)
COPD history	0.906	0.0001	2.476 (1.556-3.939)
Diabetes mellitus history	0.492	0.0152	1.636 (1.099-2.433)
Kidney dysfunction, eGFR <60 ml/min	0.590	0.0049	1.804 (1.196-2.721)

However, in a similar analysis limited only to the patients treated with nitrates, post-elective PCI mortality was not influenced by traditional mortality risk factors, including decreased LV ejection fraction, CHF, ventricular arrhythmia, diabetes, or kidney dysfunction. In nitrate-treated patients, only advanced age, decreased body mass index, and increased atherosclerosis burden, including 3-vessel CAD and left main CAD, continued to be associated with increased post-PCI mortality (Table 6).

**Table 6:** Multivariate logistic regression prediction model of in-patient mortality in patients on nitrate therapy CHF - congestive heart failure; COPD - chronic obstructive pulmonary disease; eGFR - estimated glomerular filtration rate; LM - left main; CAD - coronary artery disease

N=6,172	Mortality rate = 0.535%		
Risk factor	Coefficient	p-value	OR (95% CI for OR)
Coronary anatomy, as compared to any 1-vessel obstructive disease excluding LM			
2-vessel disease excluding LM	1.232	0.0632	3.428 (0.934-12.579)
3-vessel disease excluding LM	1.920	0.0028	6.820 (1.935-24.034)
LM and any other disease	2.375	0.0022	10.747 (2.348-49.196)
Age greater than 80 years old	1.521	0.0001	4.577 (2.108-9.939)
Weight, as compared to normal BMI 18.5-24.9 kg/m^2^			
Underweight, BMI <18.5 kg/m^2 ^	1.862	0.0334	6.438 (1.158-35.811)
Overweight, BMI 25-29.9 kg/m^2^	0.391	0.4476	1.478 (0.539-4.055)
Obese, BMI ≥ 30 kg/m^2^	0.440	0.3974	1.552 (0.561-4.298)
Left ventricular ejection fraction, as compared to normal ejection fraction >55%			
<35%	0.608	0.2047	1.836 (0.718-4.699)
35-44%	0.346	0.5405	1.414 (0.467-4.283)
45-54%	-0.231	0.6920	0.794 (0.254-2.487)
Current CHF	0.684	0.1067	1.982 (0.863-4.553)
Malignant ventricular arrhythmia	1.182	0.3106	3.262 (0.332-32.085)
COPD history	0.795	0.0690	2.215 (0.940-5.222)
Diabetes mellitus history	0.533	0.1682	1.705 (0.798-3.641)
Kidney dysfunction, eGFR <60 ml/min	0.527	0.2039	1.693 (0.751-3.817)

## Discussion

In this large, all-inclusive state-wide contemporary cohort of SIHD patients, treatment with nitrates, but not beta-blockers, calcium channel blockers, or ranolazine, was associated with increased post-PCI mortality. Furthermore, in nitrate-treated patients undergoing elective PCI, traditional predictors of mortality like decreased LV ejection fraction, ventricular arrhythmia, chronic obstructive lung disease, kidney dysfunction, diabetes, or renal insufficiency were less important, with the exception of CAD burden, advanced age, and decreased BMI.

Most patients with coronary arteriosclerosis have stable ischemic heart disease [1] and, in accord with 2002 Chronic Stable Angina Guideline and 2007 Focused Update of the American College of Cardiology (ACC)/American Heart Association (AHA) 2002 Guideline for the Management of Patients With Chronic Stable Angina, should be treated with OMT including anti-platelet agents, beta-adrenergic antagonists, ACE inhibitors or ARBs, and statins [2,3]. In SIHD angina management, first-choice drugs include beta-adrenergic blockers, calcium-channel blockers, and nitrates, while ivabradine, nicorandil, ranolazine, and trimetazidine are reserved for patients who have contraindications to the first-choice agents or remain symptomatic despite them [4,9]. Recent European guidelines suggest a patient-tailored approach which generally includes beta-adrenergic blocker and calcium channel blocker, followed with nitrates and other anti-anginal medications [14]. In patients whose symptoms are not well controlled on medical therapy, revascularization is performed for angina relief [8]. Unfortunately, despite the known benefit and higher mortality associated with nonuse, OMT for SIHD has not been consistently applied, instead favoring coronary revascularization [15,16]. To improve OMT utilization in patients with SIHD undergoing elective PCI for angina management, 2009 Appropriateness Criteria for Coronary Revascularization required maximal anti-anginal medical therapy with at least two classes of therapies [17]. More recently, thanks to the landmark Clinical Outcomes Using Revascularization and Aggressive Drug Evaluation (COURAGE) and International Study of Comparative Health Effectiveness With Medical and Invasive Approaches (ISCHEMIA) trials, the cornerstone of SIHD management has conclusively moved away from the early percutaneous coronary revascularization and instead positioned optimal medical therapy at the forefront of the treatment modalities [18,19].

However, after decades of clinical experience and guideline endorsement, there are many unresolved questions with regards to anti-anginal therapy [6,20]. It remains unclear whether a combination of the drugs or any particular drug is superior to others [5]. Anti-anginal effects of nitrates have been known since the 18th century [6-8], but the evidence of mortality benefit with nitrate SIHD therapy is lacking, and some data even suggests possible harm from using nitrates in SIHD patients [11-13]. Acting through activation of soluble guanylyl cyclase and release of nitric oxide, organic nitrates dilate veins and large and medium-sized coronary arteries and collaterals, without a significant effect on smaller arteries and capillaries [21-23]. In addition to vasodilation, nitric oxide has been shown to reduced platelet aggregation and adhesion [24]. Perplexing, despite these salutary hemodynamic and anti-platelet effects, nitrates have failed to demonstrate measurable survival benefit when used in patients with CAD.

The Gruppo Italiano per lo Studio della Sopravvivenza nell'Infarto [GISSI]-3 study investigated six-week and six-month mortality and ventricular function in 13,394 otherwise stable patients with acute ST-elevation myocardial infarction treated with lisinopril and transdermal nitroglycerin randomized in a 2x2 factorial design [25]. While lisinopril treatment led to improved survival, a decline in ventricular arrhythmia affected, and reduced LV dilation, nitrate therapy was not associated with similar benefits [25]. An observational Multicenter Study of Myocardial Ischemia (MSMI) conducted in the United States, Canada, Israel, and Japan studied mortality in 1,042 patients with documented myocardial infarction (MI) or unstable angina [11]. After an average of 26 months follow-up, nitrate therapy was associated with significantly increased mortality (4.2% vs. 0.9%, p=0.001) [11]. In addition to nitrate treatment, important MSMI mortality predictors included advanced age, previous myocardial infarction, and angina one month prior to the baseline non-invasive ischemia testing [11]. The Multicenter Diltiazem Postinfarction Trial (MDPIT) conducted in the United States and Canada evaluated the secondary preventive effect of diltiazem on mortality rate and reinfarction after MI in 2,466 patients with previous myocardial infarction [26]. After an average of 25 months follow-up, nitrate therapy was also associated with significantly increased mortality (8.7% vs. 5.0%, p=0.002) [11]. Additional MDPIT mortality predictors included age, male gender, previous MI, CHF, and diabetes [11]. Furthermore, in a prospective randomized study of 1,002 patients with a history of myocardial infarction, patients randomly assigned to nitrates had significantly increased (6.6% vs. 3.1%, p<0.05) rate of recurrent myocardial infarction, sudden death, or death from congestive heart failure [12]. In a separate small post-PCI trial of diabetic patients, nitrate therapy has also been associated with increased (26.1% vs. 6.5%, p=0.01) need for repeated revascularization, nonfatal myocardial infarction, or cardiovascular death [27]. However, these observations were made in the pre-OMT era of SIHD management. It was surprising to find that increased mortality was associated with nitrate therapy in a large contemporary cohort of PCI-treated patients on OMT.

In our cohort, nitrates were used more commonly in older patients with decreased LV ejection fraction, histories of stroke, PVD, CHF, ventricular arrhythmia, COPD, diabetes, kidney dysfunction, and more complex coronary anatomy. Thus, as suggested by other investigators, nitrate therapy may be an indicator of more advanced coronary disease and higher overall risk portending worse outcomes [28,29]. From a practical perspective, nitrates are added only in patients with continuing symptoms despite anti-platelet therapy, beta-adrenergic blockers, statins, and angiotensin-converting enzymes inhibitors. Therefore, in the modern OMT era, nitrate-treated patients are a highly selected group of patients who symptomatically "failed" OMT. Interestingly, as our analysis suggests, in nitrate-treated patients, post-elective PCI mortality is not influenced by traditional mortality risk factors, such as decreased LV ejection fraction, CHF, ventricular arrhythmia, diabetes, or kidney dysfunction. In nitrate-treated patients, only advanced age, decreased body mass index, and increased atherosclerosis burden continued to be associated with increased post-PCI mortality.

Reasons for increased mortality in nitrate-treated SIDH patients are unclear. One possibility is the nitrate tolerance phenomenon which has been well described [23]. The mechanism of nitrate tolerance is multi-faceted and includes early neurohormonal activation of vasoconstriction and intravascular volume expansion to counter nitrate-induced vasodilation and downregulation of nitro-vasodilator responsiveness with chronic nitrate therapy, endothelial dysfunction [23]. It is possible that similar mechanisms put SIHD patients treated with nitrates at a disadvantage. It is also possible that more advanced SIHD are placed on nitrates only after failing other anti-anginal therapy, as recommended in the guidelines [7]. Therefore, nitrate-treated patients may represent a special cohort of patients whose symptoms did not respond to the usual beta-adrenergic and calcium channel blocker combination. Most likely is that utilization of nitrate therapy is likely reflective of advanced disease burden, as opposed to a direct relationship between poor outcomes and this medication class alone.

Many questions in SIHD therapy remain unanswered, and new discoveries are being made. For example, more intensive blood pressure lowering in elevated risk cohorts led to a significant reduction in cardiovascular death in the SPRINT trial [30]. Sodium-glucose transport protein 2 (SGLT-2) inhibitors in patients with type 2 diabetes at high cardiovascular risk have been shown to prevent adverse cardiovascular outcomes [10]. Effects of aggressive blood pressure lowering, proprotein convertase subtilisin/kexin type 9 (PCSK9) inhibitors, and SGLT-2 on PCI outcomes are unknown. The medical therapy in SIHD continues to evolve. The question about nitrate benefit or harm would not be possible to answer without a large modern trial of nitrate therapy in SIHD patients undergoing PCI. However, as prior studies indicated and as our analysis suggests, nitrate-treated patients may represent a unique group of patients with an increased incidence of adverse outcomes. From a practical perspective, providers should be aware of an increased risk of an adverse outcome in a patient who continues to experience anginal symptoms while on OMT and requires escalation of the anti-antianginal therapy to include nitrate treatment.

The strengths and weaknesses of our study stem from the robust and extensively validated NYS PCI registry, which allowed us an opportunity to study this important subject. The database is state-wide and includes patients from all strata of the society, race, gender, and economic status. The endpoints are well defined, and the reporting is standardized. However, the patients are not randomized to the treatment categories, and the pre-procedural medical treatment was selected by the referring practitioners based on the clinical need. All patient baseline characteristics and study endpoints are limited to the ones captured and are as defined in this database. Furthermore, as we have used NYS PCI registry data, our findings cannot be extrapolated on the conservatively treated patients who are also receiving nitrate therapy. Also, the long-term outcomes of nitrate therapy in revascularized and conservatively treated patients are unknown. However, these limitations present an opportunity for future research and do not affect the findings of our study, which specifically looked at the association of nitrate therapy and in-hospital elective PCI outcomes.

## Conclusions

We investigated the effects of nitrate therapy on PCI outcomes in a large contemporary cohort of patients with SIHD. We have found that increased mortality associated with nitrate therapy in over a decade old studies continues in the current era of OMT and technologically advanced aggressive coronary revascularization. Adverse outcomes noted in nitrate-treated patients undergoing elective PCI are probably not due to the effect of nitrates but rather reflect the increased burden of co-morbidities, including older age, decreased body mass index, and increased atherosclerosis burden, including 3-vessel CAD and left main CAD in these vulnerable patients. Additional studies investigating optimal anti-anginal medical therapy on PCI outcomes are warranted.
